# Pectin modifications promote haustoria development in the parasitic plant *Phtheirospermum japonicum*

**DOI:** 10.1093/plphys/kiad343

**Published:** 2023-06-13

**Authors:** Martina Leso, Anna Kokla, Ming Feng, Charles W Melnyk

**Affiliations:** Department of Plant Biology, Linnean Center for Plant Biology, Swedish University of Agricultural Sciences, Almas allé 5, 756 51 Uppsala, Sweden; Department of Plant Biology, Linnean Center for Plant Biology, Swedish University of Agricultural Sciences, Almas allé 5, 756 51 Uppsala, Sweden; Department of Plant Biology, Linnean Center for Plant Biology, Swedish University of Agricultural Sciences, Almas allé 5, 756 51 Uppsala, Sweden; Department of Plant Biology, Linnean Center for Plant Biology, Swedish University of Agricultural Sciences, Almas allé 5, 756 51 Uppsala, Sweden

## Abstract

Parasitic plants are globally prevalent pathogens with important ecological functions but also potentially devastating agricultural consequences. Common to all parasites is the formation of the haustorium which requires parasite organ development and tissue invasion into the host. Both processes involve cell wall modifications. Here, we investigated a role for pectins during haustorium development in the facultative parasitic plant *Phtheirospermum japonicum*. Using transcriptomics data from infected Arabidopsis (*Arabidopsis thaliana*) and rice (*Oryza sativa*), we identified genes for multiple *P. japonicum* pectin methylesterases (PMEs) and their inhibitors (PMEIs) whose expression was upregulated by haustoria formation. Changes in *PME* and *PMEI* expression were associated with tissue-specific modifications in pectin methylesterification. While de-methylesterified pectins were present in outer haustorial cells, highly methylesterified pectins were present in inner vascular tissues, including the xylem bridge that connects parasite to host. Specifically blocking xylem bridge formation in the haustoria inhibited several *PME* and *PMEI* genes from activating. Similarly, inhibiting PME activity using chemicals or by overexpressing *PMEI* genes delayed haustoria development. Our results suggest a dynamic and tissue-specific regulation of pectin contributes to haustoria initiation and to the establishment of xylem connections between parasite and host.

## Teaser

The parasitic plant *Phtheirospermum japonicum* modifies the pectin methylesterification status in its haustoria in a dynamic and tissue-specific manner to allow efficient infection of the host plant.

## Introduction

Parasitic plants, which constitute around 1% of angiosperm species (Westwood et al. 2010), are important contributors to ecological systems but also include devastating pests that cause major agricultural losses each year (Rodenburg et al. 2016). Parasitism has evolved independently at least 12 times (Nickrent 2020), and despite these diverse origins, all parasitic plants form an invasive structure, the haustorium, which penetrates the host and allows the uptake of nutrients, hormones and signalling molecules (Birschwilks et al. 2006; Spallek et al. 2017; Shahid et al. 2018; Liu et al. 2020). The development of the haustorium starts with the perception of a suitable host through haustorium inducing factors (HIFs). Treatment with 2,6-Dimethoxybenzoquinone (DMBQ), the first discovered HIF, is sufficient to induce the formation of pre-haustoria in the parasitic plant family Orobanchaceae, even in the absence of a host (Chang et al. 1986). Other HIFs include hormones like cytokinin and lignin-related compounds (Goyet et al. 2017; Cui et al. 2018; Aoki et al. 2022). In the facultative root parasite *Phtheirospermum japonicum* the perception of HIFs is mediated by leucine-rich-repeat receptor-like kinases (Laohavisit et al. 2020) and increases auxin polar transport and auxin biosynthesis. The auxin signalling peak promotes cell expansion and division, leading to the formation of a swelling called the pre-haustorium (Ishida et al. 2016; Wakatake et al. 2020). Penetration of the host by the pre-haustorium is thought to depend on haustorium-secreted cell-wall-modifying enzymes such as expansins and peroxidases that loosen the host cell walls (Losner-Goshen et al. 1998; Veronesi et al. 2007; Honaas et al. 2013; Olsen et al. 2016). The invasion of the host tissues is then mediated by the intrusive cells, which differentiate from epidermal cells at the parasite-host interface and drive haustorial growth towards the host vasculature (Heide-Jørgensen and Kuijt 1995; Hood et al. 1998). Finally, a vascular connection develops between the parasite and the host. All parasitic plants form a xylem connection, which begins its differentiation from the cambium-like tissue at the centre of the haustorium (Wakatake et al. 2018). A mass of xylem tissue then develops close to the parasite vasculature (plate xylem) before strands of xylem (xylem bridges) differentiate to connect the xylem of the parasite to the xylem of the host.

Despite recent advances in our understanding of haustorium development, the mechanisms regulating haustoria initiation and host invasion remain largely unknown, but likely rely in part on cell wall modifications. In plants, lateral organ development relies on the fine tuning of cell wall modifications which are required for cell expansion, division and differentiation. These processes all require the modification of cell-to-cell adhesion. The main mediator of cell adhesion in plants is pectin, a jelly-like matrix composed of homogalacturonan, rhamnogalacturonan I and rhamnogalacturonan II (Pelloux et al. 2007; Daher and Braybrook 2015). Homogalacturonan is secreted to the cell wall in a highly methylesterified state and is then modified in the cell wall by different families of pectin modifying enzymes including pectin methylesterases (PMEs), PME inhibitors (PMEIs), polygalacturonases (PGs) and pectate lyases (PLs). Highly methylesterified pectin forms a tight matrix with less elastic properties. During developmental processes such as tissue expansion and lateral organ emergence, homogalacturonans undergo de-methylesterification by PMEs to become looser (Pelloux et al. 2007; Daher and Braybrook 2015). In addition to the fundamental roles of PMEs and PMEIs in plant development, many plant pathogens hijack plant pectin modification mechanisms to allow tissue intrusion (Hewezi et al. 2008; Raiola et al. 2011). For example, the cyst nematode *Heterodera schachtii* secretes into Arabidopsis (*Arabidopsis thaliana*) a cellulose binding protein (CBP) that activates PME3, facilitating the entry of the nematode (Hewezi et al. 2008). *A. thaliana pme3* mutants are less susceptible to nematode infection (Hewezi et al. 2008). Furthermore, some nematodes and fungi can directly secrete PMEs that mimic plant PMEs and facilitate host tissue invasion (Valette-Collet et al. 2003; Vicente et al. 2019). Parasitic plants are thought to secrete cell-wall modifying enzymes (CWMEs) to promote growth of the haustorium, adhesion to the host and loosening of host tissues to allow for invasion (Veronesi et al. 2007; Honaas et al. 2013; Yang et al. 2015; Kurotani et al. 2020). The secretion of CWMEs has been partially investigated in some parasitic plant species. For example, different species from the *Orobanche* genus secrete PMEs, PGs, PLs and peroxidases close to the site of infection to modify the host's cell wall (Ben-Hod et al. 1993; Losner-Goshen et al. 1998; Veronesi et al. 2007), while the shoot parasite *Cuscuta* upregulates *PMEI* transcription important for host penetration and increases pectin degrading enzyme activity during infection (Nagar et al. 1984; Jhu et al. 2022).

Even though cell wall modifications are suggested to be crucial for haustorium development, this aspect remains largely unexplored. Here, we use a combination of transcriptomic and genetic approaches to identify PMEs and PMEIs relevant for haustoria formation. We go on to describe dynamic and tissue specific changes in pectin methylesterification and show the effects of perturbing host and parasite PME-related enzymes. Together, this study describes the role of pectin during parasitic plant infection and reveals the importance of pectin methylesterification-related genes for haustoria development.

## Results

### *PjPMEs* and *PjPMEIs* are differentially expressed during haustorium development

**Figure 1.**
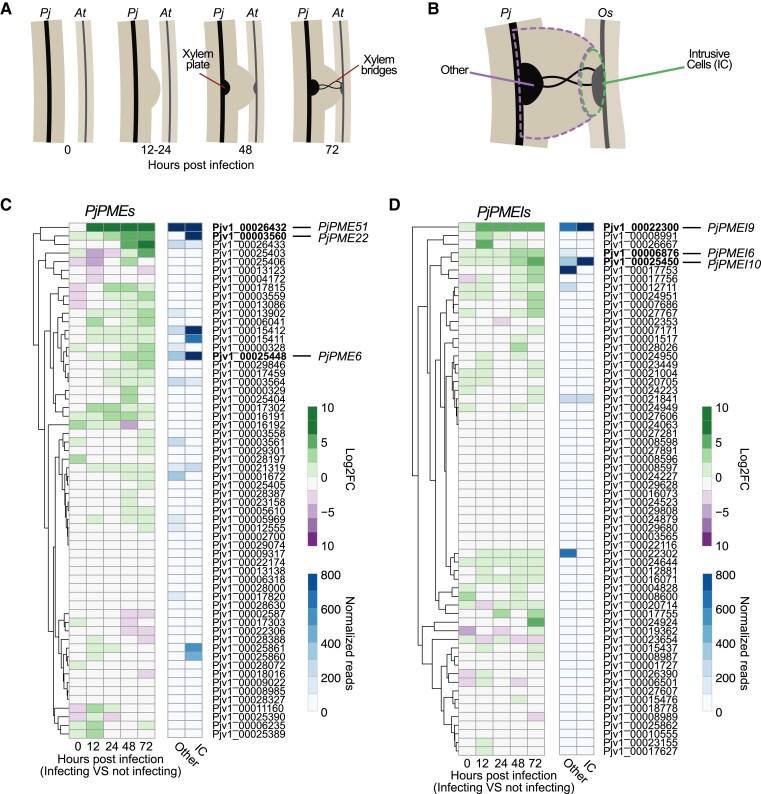
*PjPMEs* and *PjPMEIs* are differentially expressed during haustorium development. **A)** Illustration of *P. japonicum* (*Pj*) haustorium development during infection of *A. thaliana* (*At*) corresponding to the time points selected for the time course RNA-Seq (Kokla et al. 2022). **B)** Illustration of the sampling for the *P. japonicum* intrusive cells (IC) or rest of the haustorial tissues (other) during *O. sativa* (*Os*) infection for the RNA-Seq dataset presented in Ogawa et al. 2021. **C-D)** Heatmaps of the expression of candidate *P. japonicum PMEs* (*PjPMEs*) or *PMEIs* (*PjPMEIs*): log2 fold change (FC) between *P. japonicum* infecting and not infecting over five time points during infection, and normalized reads in intrusive cells and other haustorial tissue. The genes are clustered by expression according to the time-course dataset.

To study the role of pectin in *P. japonicum*, we focused on modifications of pectin by the pectin methylesterase (PME) and PME-inhibitor (PMEI) enzyme families. We performed a Hidden-Markov-Model search on the *P. japonicum* (Pj) proteome (Cui et al. 2020) and identified 73 putative PjPMEs and 62 putative PjPMEIs (Supplemental Table S1). We further filtered PjPMEs based on the presence of at least three of the five conserved catalytic amino acids (Johansson et al. 2002; Markovic and Janecek 2004) and retained 60 PjPMEs for downstream analyses (Supplemental Table S1). We aligned PjPMEs and PjPMEIs with *A. thaliana* PMEs and PMEIs, respectively, and built two Maximum-Likelihood phylogenetic trees. The trees showed co-clustering of *A. thaliana* and *P. japonicum* sequences, suggesting conservation in PMEs and PMEIs between parasite and host (Supplemental Fig. S1, A and B). We then looked at the expression of *PjPMEs* and *PjPMEIs* in two different published *P. japonicum* transcriptomic datasets. The first dataset sampled tissues at the site of haustorium development during a time-course infection of *A. thaliana* (Fig. 1A, Kokla et al. 2022), while the second dataset sampled intrusive cells (ICs) and non-ICs in mature haustoria infecting rice (*Oryza sativa*) (Fig. 1B, Ogawa et al. 2021). RNA levels of several *PjPME* and *PjPMEI* genes increased during haustorium formation, particularly at 48 and 72 h post infection (hpi), while very few genes showed decreased RNA levels (Fig. 1, C and D). Many *PjPME* and *PjPMEI* genes were also highly expressed during rice infection, including several in common with *A. thaliana* infection. We observed a greater number of highly expressed *PjPMEs* (>400 reads) in ICs compared to non-IC tissues, while several *PjPMEIs* were highly expressed in both IC and non-IC tissues (Fig. 1, C and D). In the *A. thaliana* host dataset, few *AtPMEs* and *AtPMEIs* changed expression during haustorium development with *AT1G23200* (*PME*) and *AT2G01610* (*PMEI*) showing the most consistent pattern of increased expression over multiple time points (Supplemental Fig. S1, C and D).

### PME activity increases during haustorium development and is higher in intrusive cells

**Figure 2.**
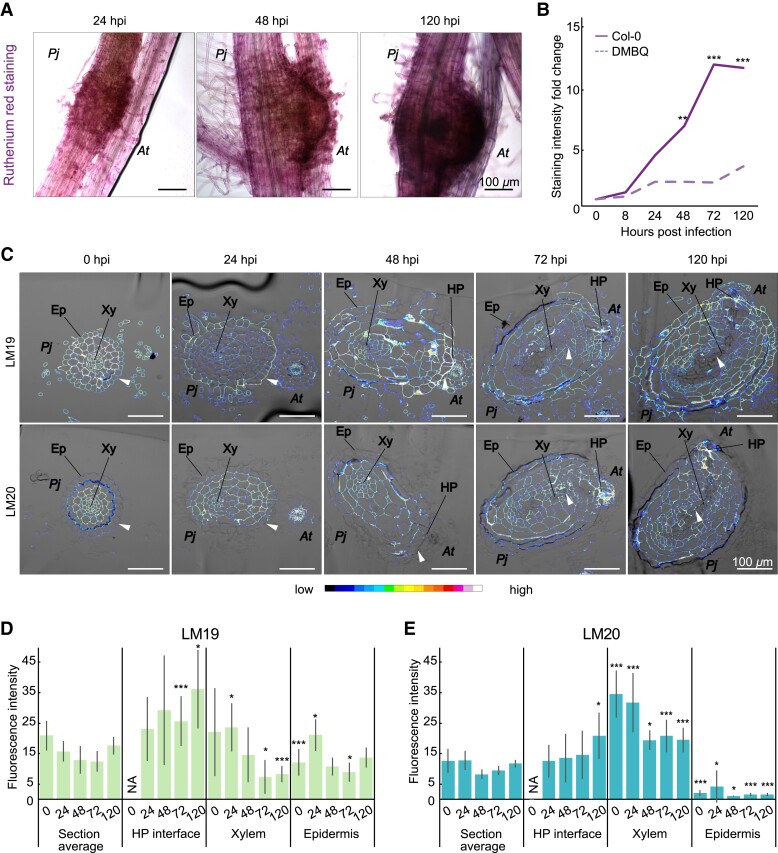
PME activity increases during haustorium development and is higher in intrusive cells. **A)** Ruthenium red staining of the developing haustoria at 24, 48 and 120 h post infection (hpi). Scale bars 100 *μ*m. **B)** Quantification of staining intensity in infecting haustoria (solid line) or pre-haustoria formed on DMBQ (dashed line) during a time course from 0 to 120 hpi. Staining intensity normalized to 0 hpi. Asterisks indicate significant difference in staining intensity between haustoria and pre-haustoria at the same time point. (** *P* < 0.01, *** *P* < 0.001, Wilcoxon test, *n* = 11 to 20 roots, 1 replicate). **C)** Fluorescence images of antibody staining with LM19 (unmethylesterified homogalacturonan) and LM20 (highly methylesterified homogalacturonan) on *P. japonicum* (*Pj*) haustoria cross sections at 0, 24, 48, 72 and 120 hpi of *A. thaliana* (*At*). Arrowheads denote areas of differential staining between LM19 and LM20. Ep = epidermis, Xy = xylem, HP = host-parasite interface. **D, E)** Fluorescence quantification in whole *P. japonicum* sections (section average), host-parasite (HP) interface, xylem (root xylem and xylem bridge) and epidermis tissues for LM19 and LM20 antibodies. Asterisks indicate significant difference between a specific tissue and the section average at the same time point (Student's t-test, *P*-value corrected for multiple testing), bars represent standard deviation. Scale bars 100 *μ*m; NA = tissue not present at the time point. * for *P* < 0.05, ** for *P* < 0.01, *** for *P* < 0.001, *n* = 3 to 15 sections.

Since we observed differential expression of *PjPMEs* and *PjPMEIs* during haustorium development, we tested whether pectin methylesterification levels could also be affected. We first performed ruthenium red staining on whole roots during an infection time course (Fig. 2A). Ruthenium red binds with higher affinity to de-methylesterified homogalacturonan, and therefore a higher staining often corresponds with higher PME activity (Downie et al. 1998). We observed an increase in ruthenium red staining by 24 hpi at the interface between *P. japonicum* and *A. thaliana*. The staining intensity increased further in later stages corresponding to host invasion (48 hpi) and xylem differentiation (72 and 120 hpi) (Fig. 2, A and B, Supplemental Fig. S2A), but was not observed when inducing haustoria using DMBQ (Fig. 2B, Supplemental Fig. S2B), which does not induce xylem bridge formation. We also used LM19 and LM20 antibodies specific for de-methylesterified and highly methylesterified pectin (Verhertbruggen et al. 2009), respectively, to measure pectin modifications from 0 to 120 hpi (Fig. 2, C, D and E, Supplemental Fig. S2C). LM19 staining was mostly localised to the outer epidermis and at the host-parasite interface, especially in later time points (72 and 120 hpi), whereas staining intensity was reduced in xylem tissues during infection (Fig. 2, C and D). LM20 staining was nearly absent in epidermal tissues and staining was instead mainly focused to xylem tissues and at the host-parasite interface (Fig. 2, C and E). These results showed that pectin methylesterification was modified dynamically during haustorium development in a tissue-specific manner.

### Differentially expressed *PjPMEs* and *PjPMEIs* activate in intrusive cells and cambium-like tissue

**Figure 3.**
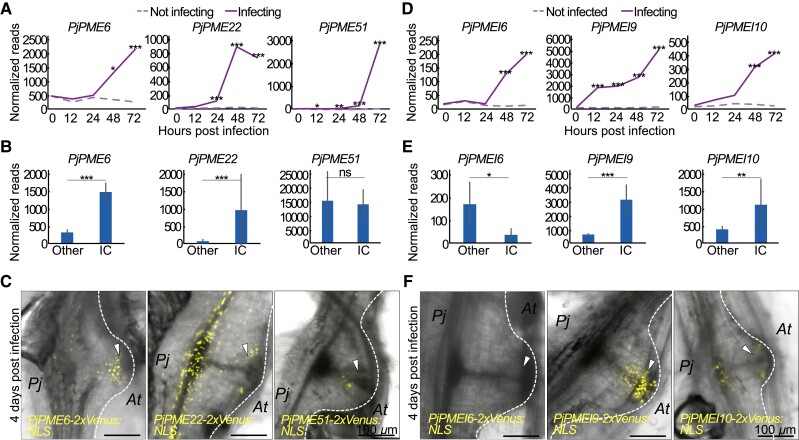
Upregulated *PjPMEs* and *PjPMEIs* are primarily expressed in intrusive cells and cambium-like tissue. **A, D)** Normalized reads of *PjPME6*, *PjPME22*, *PjPME51, PjPMEI6*, *PjPMEI9* and *PjPMEI10* over five time points during infection for *P. japonicum* infecting and not infecting. Asterisks indicate a significant difference between infecting and not infecting (Wald test with Benjamini-Hochberg correction for multiple testing, *n* = 3 libraries). **B, E)** Normalized reads of *PjPME6*, *PjPME22*, *PjPME51, PjPMEI6*, *PjPMEI9* and *PjPMEI10* in intrusive cells and non-IC (other) tissues. Asterisks indicate a significant difference between IC and other tissues (Student's t-test, *n* = 3 libraries). Bars represent standard deviation. **C, F)** Images of infecting transgenic hairy roots expressing *PjPME6*, *PjPME22*, *PjPME51, PjPMEI6*, *PjPMEI9* and *PjPMEI10* nuclear-localized (NLS) transcriptional reporters in fully developed haustoria (4 dpi). Arrowheads denote intrusive cells. Scale bars 100 *μ*m. For all panels * for *P* < 0.05, ** for *P* < 0.01, *** for *P* < 0.001.

Since the pectin degree of methylesterification (DM) in the developing haustorium was different in specific tissues, we investigated candidate *PjPMEs* and *PjPMEIs* to understand if their expression pattern corresponded with the pattern of pectin DM. We chose three *PjPME* genes with increased expression in the time course dataset and renamed them based on the *A. thaliana* homolog (Fig. 1C, Fig. 3A). All three genes were also upregulated during *P. japonicum*-*O. sativa* infection. *PjPME6* (*Pjv1_00025448*) and *PjPME22* (*Pjv1_00003560*) expression levels increased in ICs compared to other tissues, while *PjPME51* (*Pjv1_00026432*) was highly expressed in both IC and non-IC tissues (Fig. 3B). We made and transformed transcriptional reporters in *P. japonicum* hairy roots and found *PjPME6* and *PjPME51* reporters showed signal mainly in ICs, while the *PjPME22* reporter was mostly expressed in *P. japonicum* vasculature (Fig. 3C). We then selected three *PjPMEI* genes upregulated during haustorium development in *A. thaliana* and *O. sativa* infections (Fig. 1D and 3D). In the *P. japonicum*-*O. sativa* dataset, *PjPMEI9* (*Pjv1_00022300)* and *PjPMEI10 (Pjv1_00025450)* showed increased expression in ICs, while *PjPMEI6* (*Pjv1_00006876)* was more expressed in non-IC tissues (Fig. 3E). Our transcriptional reporters for these genes showed expression in ICs and cambium-like tissues for *PjPMEI9*, and plate xylem and ICs for *PjPMEI10* (Fig. 3F). The *PjPMEI6* reporter showed no fluorescence in 4 dpi haustoria (Fig. 3F). All the *PjPMEs* and *PjPMEIs* we investigated were not expressed in the primary root tip of the hairy roots, and showed little or no fluorescence at the lateral root emergence sites of the hairy roots, suggesting that upregulation of these genes was specific to haustorium development (Supplemental Fig. S3).

### Inhibition of PME activity impairs haustoria induction and development

**Figure 4.**
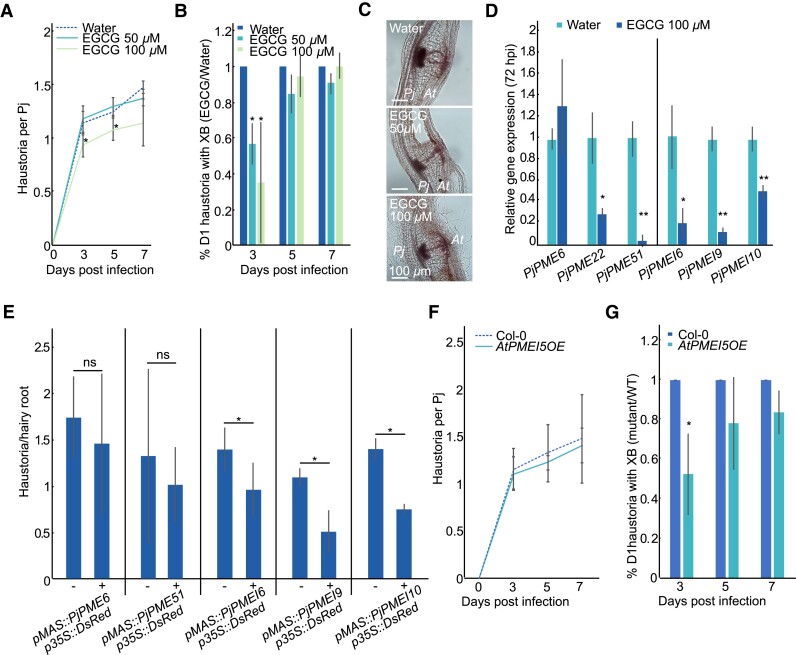
Inhibition of PME activity impairs haustorium induction and development. **A)** Number of haustoria per *P. japonicum* plant at four time points during treatment with 50 *μ*M ECGC, 100 *μ*M EGCG or water control. Asterisks indicate significance compared to control (Student's t-test, *n* = 3 replicates). **B)** Ratio of the percentage of day one (D1) haustoria with a xylem bridge (XB) formed during treatment with 50 or 100 *μ*M EGCG over water at three time points. Asterisks indicate significance compared to control (Student's t-test, *n* = 3 replicates). **C)** Images of 7 dpi haustoria formed on water, 50 *μ*M EGCG and 100 *μ*M EGCG. **D)** Relative gene expression of selected *PjPMEs* and *PjPMEIs* at 72 hpi in *P. japonicum* haustoria treated with 100 *μ*M EGCG, normalised to water. Asterisks indicate significance compared to control (Student's t-test, *n* = 3 replicates). **E)** Numbers of haustoria per hairy root transformed with *PjPME* and *PjPMEI* overexpression constructs. Non-transgenic hairy roots are marked as “-” and transgenic roots are marked as “+”. Asterisks indicate significance compared to control (Fisher's exact test; *n* = 2 to 4 replicates, 12 to 53 total roots per sample). **F)** Number of haustoria per *P. japonicum* plant at four time points during infection of *AtPMEI5OE* mutant or Col-0 as control (*n* = 3 replicates). **G)** Ratio of the percentage of D1 haustoria with a XB formed during infection of *AtPMEI5OE* over Col-0 at three time points. Asterisks indicate significance compared to control (Student's t-test, *n* = 3 replicates). For all panels * for *P* < 0.05, ** for *P* < 0.01, bars represent standard deviation.

To determine if PME activity is necessary for haustorium development, we treated infecting *P. japonicum* with 50 *µ*M or 100 *µ*M of epigallocatechin gallate (EGCG), a chemical inhibitor of PME enzymes (Lewis et al. 2008). Treatment reduced the number of haustoria (Fig. 4A) and delayed the formation of xylem bridge connections to the host (Fig. 4B), although it did not substantially affect plate xylem area and number of xylem bridge connections at 7 dpi (Fig. 4C, Supplemental Fig. S4, A and B). EGCG treatment also reduced *PjPME22*, *PjPME51* and *PjPMEI10* expression at 72 hpi (Fig. 4D), and reduced *PjPMEI6* and *PjPMEI9* expression at both 0 and 72 hpi, suggesting that chemical inhibition of PMEs affected both haustoria development and the transcriptional regulation of endogenous *PMEs* and *PMEIs* (Fig. 4D, Supplemental Fig. S4C). We next overexpressed *PjPME6* and *PjPME51* in *P. japonicum* hairy roots (Supplemental Fig. S4, D and E) but did not observe defects in haustorium induction or development (Fig. 4E, Supplemental Fig. S4, F and G). However, overexpression of *PjPMEI6, PjPMEI9* and *PjPMEI10* significantly inhibited haustoria induction (Fig. 4E) but did not affect xylem connections (Supplemental Fig. 4, F and G). Finally, we tested whether modifying pectin status in the host could affect infection by using the *PMEI5*-overexpressing *A. thaliana* line *AtPMEI5OE*, which is characterised by highly methylesterified pectin (Wolf et al. 2012; Jonsson et al. 2021). Haustoria induction was not affected in the mutant compared to wild-type Col-0 (Fig. 4F), however, xylem bridge formation was delayed during infection of *AtPMEI5OE* (Fig. 4G). Taken together, these results suggest that parasitic PME activity is important for efficient induction and development of haustoria.

### Brassinosteroid treatment reduces *PjPME* and *PjPMEI* expression and delays haustorium development

**Figure 5.**
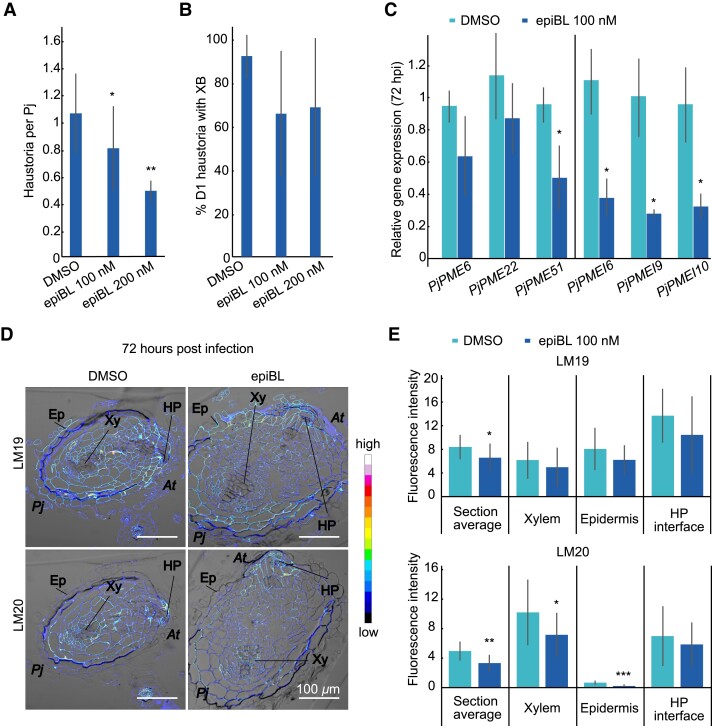
BR treatment reduces *PjPME* and *PjPMEI* expression and inhibits haustorium development. **A)** Number of haustoria per *P. japonicum* plant at 7 dpi during treatment with 100 nM epiBL, 200 nM epiBL or DMSO control (*n* = 4 to 6 replicates). **B)** Percentage of day one (D1) haustoria with a xylem bridge (XB) formed during treatment with 100 nM epiBL, 200 nM epiBL or DMSO at 7 dpi (*n* = 4 to 6 replicates). **C)** Relative gene expression of selected *PjPMEs* and *PjPMEIs* at 72 h post infection in *P. japonicum* haustoria treated with 100 nM epiBL, normalised to DMSO (*n* = 3 replicates). **D)** Fluorescence images of antibody staining using LM19 (unmethylesterified homogalacturonan) and LM20 (highly methylesterified homogalacturonan) on cross sections of 72 hpi haustoria formed on DMSO control or 100 nM epiBL. Scale bars 100 *μ*m. Ep = epidermis, Xy = xylem, HP = host-parasite interface. *At* = *A. thaliana*, *Pj* = *P. japonicum*. **E)** Fluorescence quantification in whole *P. japonicum* sections (section average), host-parasite (HP) interface, xylem (root xylem and xylem bridge) and epidermis tissues for LM19 and LM20 antibodies (*n* = 7 to 14 sections). For all panels, asterisks indicate significance compared to DMSO (Student's t-test): * for *P* < 0.05, ** for *P* < 0.01, bars represent standard deviation.

Brassinosteroid (BR) signalling mediates cell wall biosynthesis and remodelling, and has been implicated in feedback mechanisms with PME and PMEI activity and EGCG treatments (Wolf et al. 2012). To test the effect of BRs, we applied 100 nM or 200 nM of epibrassinolide (epiBL) during *P. japonicum* infection and found it reduced the number of haustoria per *P. japonicum* (Fig. 5, A and B, Supplemental Fig. S5A), similar to EGCG treatment (Fig. 4, A and , B). We also tested the expression of *PjPME*s and *PjPMEI*s in haustoria following epiBL treatment. *PjPME51*, *PjPMEI6*, *PjPMEI9* and *PjPMEI10* were downregulated in haustoria treated with epiBL at 72 hpi but not at 0 hpi (Fig. 5C, Supplemental Fig. S5B), suggesting transcriptional control of pectin methylesterification by BR signalling during haustorium development. To determine if BR treatment could modify pectin methylesterification levels, we performed antibody staining using LM19 and LM20 on cross sections of 0 hpi and 72 hpi haustoria untreated or treated with epiBL (Fig. 5D, Supplemental Fig. S5C). The haustoria treated with epiBL had lower levels of both unmethylesterified pectin (LM19) and highly methylesterified pectin (LM20) compared to the control (Fig. 5E), suggesting overall pectin levels were reduced following epiBL treatment. In particular, staining for highly methylesterified pectin (LM20) was significantly lower in xylem and epidermis tissues treated with epiBL (Fig. 5E), corresponding with the reduced *PjPMEI* gene expression previously observed (Fig. 5C). Finally, we infected the *A. thaliana BRI1-EMS-SUPPRESSOR 1* mutants with modified BR signalling, *bes1-2* and *bes1-D*, to test the role of host BR signalling during infection. *P. japonicum* could efficiently infect both mutants and establish xylem connections (Supplemental Fig. S5, D and E), suggesting host BR signalling is not crucial for haustoria development and instead BR signalling might be important for parasite cell wall modifications during infection.

### *PjPMEs* and *PjPMEIs* expression associates with xylem bridge development

**Figure 6.**
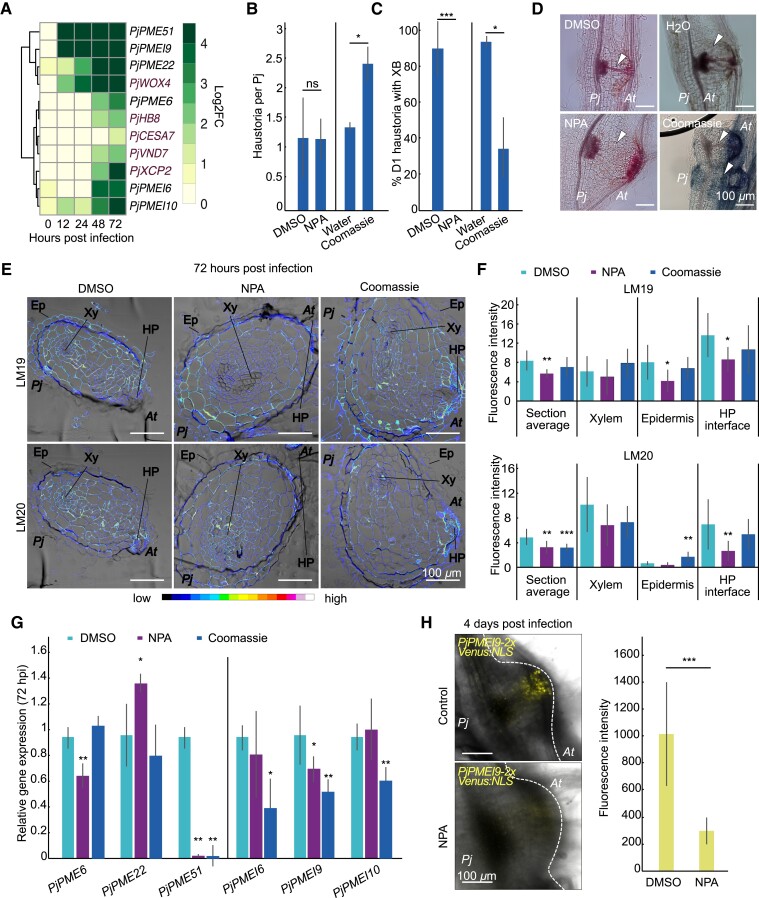
*PjPMEs* and *PjPMEIs* expression associates with xylem bridge development. **A)** Expression heatmap of *P. japonicum* cambium and xylem marker genes (red) and selected *PjPMEs* and *PjPMEIs*: log2 fold change between *P. japonicum* infecting and not infecting over five time points during infection, clustered by expression. **B)** Number of haustoria per *P. japonicum* plant at 7 dpi during treatment with DMSO, 5 *μ*M NPA (*n* = 2 replicates), water or 0.05 mM Coomassie (*n* = 3 replicates). **C)** Percentage of day one (D1) haustoria with a xylem bridge (XB) formed during treatment with DMSO, 5 *μ*M NPA (*n* = 2 replicates), water or 0.05 mM Coomassie (*n* = 3 replicates). **D)** Images of 7 dpi haustoria formed on DMSO, 5 *μ*M NPA, water or 0.05 mM Coomassie. White arrowheads denote the location of XB development. **E)** Fluorescence images of antibody staining using LM19 and LM20 on cross sections of 72 hpi haustoria developed on DMSO, 5 *μ*M NPA or 0.05 mM Coomassie + DMSO. Ep = epidermis, Xy = xylem, HP = host-parasite interface. *At* = *A. thaliana*, *Pj* = *P. japonicum*. **F)** Fluorescence quantification in whole *P. japonicum* sections (section average), host-parasite (HP) interface, xylem (root xylem and xylem bridge) and epidermis tissues for LM19 and LM20 antibodies. Asterisks indicate significance compared to DMSO (Student's t-test, *P*-value corrected for multiple testing, *n* = 8 to 14 sections). **G)** Relative gene expression of select *PjPMEs* and *PjPMEIs* at 72 hpi in *P. japonicum* haustoria treated with 5 *μ*M NPA or 0.05 mM Coomassie + DMSO normalised to DMSO (*n* = 3 replicates). **H)** Images of haustoria developed on hairy roots expressing the *PjPMEI9* nuclear-localised (NLS) transcriptional reporter at 4 dpi on DMSO or 5 *μ*M NPA, and quantification of fluorescence intensity (*n* = 7 to 10 haustoria). Scale bars 100 *μ*m. For all panels, asterisks indicate significance compared to control (Student's t-test): * for *P* < 0.05, ** for *P* < 0.01, *** for *P* < 0.001, bars represent standard deviation.

Since some *PjPMEs* and *PjPMEIs* are expressed in the cambium and xylem-like tissues during haustorium development (Fig. 3C-F) and inhibiting PME activity delays xylem formation (Fig. 4B), we investigated the role of *PjPMEs* and *PjPMEIs* during xylem-bridge formation. Looking at the expression pattern of cambium and xylem marker genes, we found the cambium marker *WUSCHEL RELATED HOMEOBOX 4* (*PjWOX4)* (Wakatake et al. 2018) co-expressed with *PjPME22*, *PjPME51* and *PjPMEI9* (Fig. 6A). The putative procambium marker *HOMEOBOX GENE 8* (*PjHB8)* co-expressed with *PjPME6*. The xylem-markers *CELLULOSE SYNTHASE A 7* (*PjCESA7)* (Wakatake et al. 2018), *VASCULAR RELATED NAC-DOMAIN PROTEIN 7* (*PjVND7*) (identified through BLAST using *AtVND7* as a query) and *XYLEM CYSTEINE PEPTIDASE 2* (*PjXCP2*) (Kokla et al. 2022) co-expressed with *PjPMEI6* and *PjPMEI10* (Fig. 6A, Supplemental Fig. 6A). To investigate if pectin methylesterification levels change in response to xylem-bridge development, we chemically inhibited xylem bridge formation by treatment with the auxin transport inhibitor N-1-naphthylphthalamidic acid (NPA). NPA treatment did not affect haustoria numbers, yet inhibited xylem bridge connection (Wakatake et al. 2020, Fig. 6, B and D). Treatment with the synthetic auxin 1-naphthaleneacetic acid (NAA) did not affect haustoria numbers or xylem bridge formation (Supplemental Fig. S6B, C and D). We also found that the commonly used dye Coomassie Brilliant Blue, an inhibitor of xyloglucan endotransglucosylase/hydrolase (XTH) activity (Olsen and Krause 2017), increased haustoria numbers, yet reduced xylem bridge formation by approximately 60% (Fig. 6B-D). LM19 and LM20 antibody staining of 72 hpi sections showed both de-methylesterified pectin and highly methylesterified pectin were reduced following NPA treatment, mostly in the host-parasite interface (Fig. 6, E and F, Supplemental Fig. S6E). Staining with LM20 was also reduced following Coomassie treatment (Fig. 6, E andF, Supplemental Fig. S6E). Expression levels of several PMEs and PMEIs were significantly affected by NPA and Coomassie treatments (Fig. 6G) including *PjPME51* and *PjPMEI9* that were decreased by both treatments at 72 hpi (Fig. 6g) but not at 0 hpi (Supplemental Fig. S6F). This decreased expression of *PjPME51* and *PjPMEI9* appeared specific to xylem bridge inhibition and suggested a role for these genes in xylem bridge formation. We then infected hairy roots expressing *PjPMEI9-2xVenus:NLS*, which showed fluorescence in cambium-like tissues (Fig. 3F, Supplemental Video S1). At 4 days post infection we observed a marked decrease in fluorescence when hairy roots expressing *PjPMEI9-2xVenus:NLS* were treated with NPA compared to the control, consistent with the RT-qPCR data (Fig. 6H) and demonstrating that xylem bridge formation is important for *PMEI9* expression.

## Discussion

Here, we investigated the role of PME-mediated pectin modifications during haustorium development in *P. japonicum* and identified multiple *PjPMEs* and *PjPMEIs* upregulated during *A. thaliana* and *O. sativa* infections. Induction dynamics varied with some genes highly activated during early stages of infection whereas others peaked late when xylem bridges formed (Fig. 1C-D and 3, A and D), suggesting they had different developmental roles as infection progressed. The reporters we generated showed PME-related gene expression in intrusive cells (Fig. 3B-C) and these outer tissues also showed low methylesterification (Fig. 2C) suggesting cell wall loosening was relevant for expansion of outer tissues and interaction with the host. Suppressing PME activity by overexpressing PMEIs or by chemical treatments with EGCG also reduced the ability for haustoria to form (Fig. 4) consistent with a role for PMEs and pectin loosening in organogenesis and haustoria expansion. Recently, it was found that pectin methylesterification levels are also crucial for lateral root initiation (Wachsman et al. 2020). Our findings suggested aspects of lateral root formation and haustoria emergence are conserved in *P. japonicum* as it has been previously suggested for *Cuscuta* and *Thesium* parasites (Ichihashi et al. 2018; Jhu et al. 2021).

We also observed strong induction of PMEI-related gene expression including in the vascular tissues of the haustoria (Fig. 1D and 3D-F). Antibody staining revealed high levels of methylesterified pectin in the inner haustoria tissues and xylem bridges (Fig. 2C-D) that could provide structural support to these tissues. Thus, the apparent co-expression of both esterase (PME) and inhibitor (PMEI) could be explained in part by differences in spatial expression and the degree of methylesterification in different tissues. Asymmetric pectin methylesterification is required in several plant developmental processes, including apical hook formation during seedling emergence and leaf patterning (Jonsson et al. 2021; Peng et al. 2022). Furthermore, differences in pectin methylesterification between lateral roots and its progenitor tissues might allow the emergence of the lateral root while preventing its own digestion (Laskowski et al. 2006). We propose that in haustoria high PME activity in intrusive cells drives host cell wall loosening and penetration, while high PMEI activity maintains inner haustorial tissue integrity and helps these structures push towards the host.

Our investigations also revealed a tight association between pectins and xylem bridge formation. DMBQ induces pre-haustoria that lack xylem bridges (Cui et al. 2016) and we observed little ruthenium red staining of DMBQ treatment samples (Fig. 2B, Supplemental Fig. S2B) suggesting that pectins were not highly de-methylesterified during pre-haustoria formation. Similarly, EGCG, NPA and Coomassie Brilliant Blue treatments all delayed or inhibited xylem bridge formation (Fig. 4B and 6C) and reduced the expression of *PME51* and *PMEI9* (Fig. 4D and 6G) suggesting a close relation between xylem bridge formation and *PME51* and *PMEI9* activation. In alfalfa (*Medicago sativa*), xylem cell walls contain about 4% pectin, compared to 25% pectin in other tissues (Grabber et al. 2002), suggesting pectin might be degraded during xylem differentiation. In *A. thaliana*, five *PMEs* are expressed in xylem tissues (Pelloux et al. 2007), and the demethylesterification of pectin might be important for lignification (Lairez et al. 2005; Pelloux et al. 2007). PME activity including from *PjPME51* might therefore be required in the first stage of xylem bridge differentiation to allow pectin degradation, followed by lignification.

In the *A. thaliana* host, our data suggest the role of pectin modifications during infection is less important. Only *AT1G23200* (*PME*) and *AT2G01610* (*PMEI*) showed clear increases in expression during infection (Supplemental Fig. S1, C and D), suggesting these genes might either be involved in a defence response to the parasite or are activated by *P. japonicum* to facilitate parasitism. Notably, the *A. thaliana* overexpressor *AtPMEI5OE* (Wolf et al. 2012), which has high methylesterification levels (Wolf et al. 2012; Jonsson et al. 2021), delayed xylem bridge connections (Fig. 4, F and G) indicating that pectin modifications by the host could influence parasite development. Our EGCG treatments likely inhibited both parasite and host PMEs so our finding that EGCG reduces xylem bridge formation could be explained in part due to inhibition of host PMEs. Thus, cell wall modifications by both host and parasite appear relevant for successful parasitic plant infection and deserve further attention. By better understanding and modifying the host cell wall response, it may be possible to achieve durable resistance to parasites.

## Materials and methods

### Plant materials and growth conditions

*Phtheirospermum japonicum* and Arabidopsis (*Arabidopsis thaliana*) seeds were surface sterilized by washing with 70% v/v ethanol for 20 min, followed by 95% v/v ethanol for 5 min, and sown on 1⁄2 MS medium with 1% w/v sucrose and 0.8% w/v bactoagar. After stratification at 4 °C in darkness for 1 or 2 days for *P. japonicum* and *A. thaliana* respectively, the plates were moved to a growth cabinet at 25 °C in long day conditions (16 h light/8 h darkness), 100 *μ*mol m^−2^ s^−1^ light. The *A. thaliana* Col-0 accession was used unless otherwise stated. The *AtPMEI5OE* line has been previously published (Wolf et al. 2012). The BR-signalling mutants *bes1-2* and *bes1-D* have been previously published (Yin et al. 2002; Lachowiec et al. 2013).

### PjPMEs and PjPMEIs identification and phylogenetic analyses

*A. thaliana* PME (Louvet et al. 2006) and PMEI (Wang et al. 2013) sequences were downloaded from the Phytozome database (Goodstein et al. 2012). *P. japonicum* putative PMEs and PMEIs were identified by searching the HMM profiles (PF01095 and PF04043 respectively) on a *P. japonicum* proteome obtained from the published genome (Cui et al. 2020) using the HMMER3 software (Finn et al. 2011). The putative PjPMEs were aligned using Clustal W in MEGAX (Tamura Stecher and Kumar 2021), and the sequences lacking more than one of the five conserved catalytic amino acids (Johansson et al. 2002; Markovic and Janecek 2004) were removed from downstream analyses. *P. japonicum* and *A. thaliana* PME and PMEI sequences were aligned using ClustalW. Maximum-Likelihood phylogenetic trees were built using MEGAX with 100 bootstraps.

### *In vitro* infection assays with *Phtheirospermum japonicum*

Infection assays were performed according to Kokla et al. 2022. Briefly, five days after germination *P. japonicum* seedlings were moved from nutrient medium to nutrient-free medium (water agar) for starvation. After three days, a six-day old *A. thaliana* seedling was aligned root-to-root to each *P. japonicum* seedling to allow infection. 50 *μ*M or 100 *μ*M EGCG, 100 nM or 200 nM epiBL, 5 *μ*M NPA, 0.05 mM Coomassie Brilliant Blue and 0 to 500 nM NAA were applied directly in the nutrient-free medium and left until the end of the infection period. For measuring the plate xylem area and the number of xylem bridges, 7 days post infection (dpi) haustoria were stained with Safranin-O following the method in Spallek et al. 2017. Pictures were taken using an Axioscope A1 microscope and analysed in Fiji (Schindelin et al. 2012; Rueden et al. 2017).

### Immunohistochemical staining of pectin residues

*P. japonicum* infecting *A. thaliana* was harvested at 0, 24, 48, 72 and 120 hpi for infections on water. For infections on DMSO, 100 nM epiBL (dissolved in DMSO), 0.05 mM Coomassie (plus DMSO) and 5 *μ*M NPA (dissolved in DMSO) treated samples were harvested and at 0 and 72 hpi. The seedlings were fixed in a 1% v/v glutaraldehyde, 4% w/v formaldehyde, 0.05 M NaPi aqueous solution by vacuuming twice for 20 min, followed by overnight incubation at 4 °C. The samples were then dehydrated with an ethanol gradient (30 min in each of 10%, 30%, 50%, 70%, 96%, 100%, 100% v/v ethanol) and incubated overnight in a 1:1 solution of 100% ethanol:Historesin solution (Leica). The solution was exchanged with Historesin and the samples incubated again overnight at 4 °C. The seedlings were then oriented in molds following the method in (Scheres et al. 1994), aligning the haustoria of different seedlings. The shoot was removed, and a 14:1 solution of Historesin and hardener was added to form a hard resin sheet. The haustoria were cross-sectioned at 8 *μ*m thickness using a Microm HM355 S microtome. The sections were rehydrated in PBS, incubated in 0.05 M glycine in PBS for 20 min, and blocked in 2% w/v BSA in PBS (blocking buffer) for 30 min. Three consecutive slides were stained in 1:20 dilutions of LM19, LM20 in PBS or just PBS for the negative control and incubated for 2 h. After rinsing with blocking buffer three times, the sections were incubated for 1 h in a 1:100 dilution of Goat anti-Rat IgG Alexa Fluor 647 secondary antibody. After rinsing three times with PBS, the sections were mounted in PBS and immediately imaged on a Zeiss LSM-780 confocal microscope with 633 nm excitation, 0.5% laser power, 650 gain and 633 to 695 nm detection. Fluorescence was quantified in Fiji using the mean gray value measurement after selecting the area corresponding to the desired tissue on the brightfield channel. Three to fifteen different haustoria were imaged and quantified for each time point and treatment. The same DMSO control was used for epiBL presented in Fig. 5 and Coomassie and NPA presented in Fig. 6. Representative images were processed equally for each panel using the 16-colors LUT to allow easier visualization of fluorescence intensity.

### Ruthenium red staining

Ruthenium red staining was performed by dipping infecting roots at 0, 24, 48, 72 and 120 hpi in 0.05% w/v ruthenium red in deionised water for 5 min, followed by rinsing 2 times with deionised water and mounting on 20% v/v glycerol. Pictures were taken using an Axioscope A1 microscope and analysed in Fiji (Schindelin et al. 2012; Rueden et al. 2017).

### RNA-Seq datasets and gene accession numbers

The RNA-seq dataset used for gene expression analyses of the infection time course in *P. japonicum* and *A. thaliana* is presented in Kokla et al. 2022. The dataset used for gene expression analyses in intrusive and non-intrusive haustorial cells is presented in Ogawa et al. 2021. The heatmaps were generated using the “pheatmap” function in RStudio on the log2 fold change (time-course dataset) or normalised reads (IC vs non-IC dataset) of the genes indicated in each heatmap. The heatmaps were clustered by expression in the time course dataset. Genes IDs and accession numbers for the *P. japonicum* genes mentioned in the text are available in Supplemental Table S2.

### Gene expression analyses

Forty 5-day-old *P. japonicum* seedlings per biological replicate per treatment were transferred to the starvation medium with 100 *μ*M EGCG, 100 nM epiBL, 5 *μ*M NPA, 0.05 mM Coomassie, or 1 *μ*M NAA for three days or control DMSO. After infecting *A. thaliana*, 2 mm of root around the haustorium was collected at 0 or 72 h post infection. RNA was extracted using the ROTIPrep RNA MINI kit (Carl Roth, 8485) following the manufacturer's instructions. cDNA was synthesised with the Maxima First Strand cDNA Synthesis Kit for RT-qPCR (ThermoFisher, K1642) following the manufacturer's instructions. RT-qPCR was performed using the Maxima SYBR Green/ROX qPCR Master Mix 2x (Thermo Scientific, K022). *P. japonicum SERINE/THREONINE PROTEIN PHOSPHATASE 2A* (*PjPP2A*) was used as normalisation control (Serivichyaswat et al. 2022). For each experiment, three biological replicates and at least two technical replicates were used. The relative gene expression was calculated using the Pfaffl method. The primers used are available in Supplemental Table S3.

### Cloning of *PjPMEs* and *PjPMEIs* and plasmid construction

All cloning was based on the Greengate cloning method following the standard protocols (Lampropoulos et al. 2013). Greengate plasmids used for cloning have been previously published (Lampropoulos et al. 2013). All primers used for GreenGate cloning are listed in Supplemental Table S3. Digestion and ligation reactions were performed using the BsaI-HFv2 (NEB #R3733) and T4 DNA Ligase (NEB M0202) enzymes respectively. For the overexpression constructs, the CDS of *PjPME6, PjPME51*, *PjPMEI9, PjPMEI6* and *PjPMEI10* were amplified using the CloneAmp HiFi PCR Premix (TakaraBio) from the cDNA of *P. japonicum* and inserted into the entry vector pGGC000 (Addgene plasmid # 48858) to yield pGGC-CDS vectors. The ligated plasmids were amplified in chemically competent *Escherichia coli* DH5α and confirmed by Sanger sequencing. The final binary vector assembly was performed using pGGA-pMAS, pGGB003 (Addgene plasmid # 48821), pGGC-CDS, pGGD002 (Addgene plasmid # 48834), pGGE-terMAS, pGGF-DsRed and pGGZ001 (Addgene plasmid # 48868). pGGA-pMAS, pGGE-terMAS and pGGF-DsRed were previously published (Kokla et al. 2022). For the reporter constructs, a sequence of ∼3 kb upstream the starting codon was cloned as the promoter of the genes of interest (pGOI). *PjPME6* (3087 bp), *PjPME51* (2876 bp), *PjPME22* (3044 bp), *PjPMEI9* (3021 bp), *PjPMEI10* (3071 bp) and *PjPMEI6* (2988 bp) promoters were amplified using the CloneAmp HiFi PCR Premix (TakaraBio) from the gDNA of *P. japonicum* and inserted into the entry vector pGGA000 (Addgene plasmid # 48856) to yield pGGA-pGOI vectors. A 2xVenus-NLS sequence was cloned from a previously published GoldenGate vector backbone (Cui et al. 2016) and inserted in the pGGC000 entry vector to create pGGC-2xVenus-NLS. The ligated plasmids were amplified in chemically competent *E. coli* DH5α and confirmed by Sanger sequencing. The final binary vector assembly was performed using pGGA-pGOI, pGGB003, pGGC-2xVenus-NLS, pGGD002, pGGE001 (Addgene plasmid # 48839), pGGF-DsRed and pGGZ001. The final overexpression and reporter plasmids were co-transformed in electrocompetent *Agrobacterium rhizogenes* AR1193 with the pSoup plasmid (Addgene plasmid # 165419), and the bacteria cultured in LB broth with 50 *μ*g/ml spectinomycin and 50 *μ*g/ml rifampicin.

### *P. japonicum* hairy root transformation

*P. japonicum* transformation was performed according to Ishida et al. 2011. Seven-day-old *P. japonicum* seedlings were sonicated for 10 s and vacuum-infiltrated for 5 min in a solution of AR1193 carrying the construct of interest. The seedlings were then moved to solid B5 medium supplemented with 1% w/v sucrose and 450 *μ*M acetosyringone and kept at 22 °C in the dark for 2 days. Seedlings were then moved to B5 medium containing 300 *μ*g/ml cefotaxime and grown at 25 °C in long day conditions until formation of hairy roots. Transgenic hairy roots were identified through red fluorescence using a Leica M205 FA stereo microscope and placed on starvation medium for 4 days before addition of *A. thaliana*. Non-fluorescent hairy roots from the same transformation experiment were used as a control for each construct. Counting of haustoria and safranin-O staining were performed at 7 dpi for overexpression constructs. Imaging of transcriptional reporters was performed on 4 dpi haustoria using a Zeiss LSM780 confocal microscope with 514 nm excitation, 2.8% laser power, 950 gain and 519 to 550 nm detection.

### Statistics

All experiments were replicated at least three times unless otherwise stated. For infection assays each biological replicate consisted of the average of results from at least 15 plants, and one-tailed Student's t-tests on means were used for single comparisons. For assays with transformed hairy roots overexpressing *PjPMEs* or *PjPMEIs*, the data from the biological replicates were pooled and divided in categories of 0, 1 or ≥2 haustoria per hairy root. A Fisher exact test was then used to calculate significance. For RT-qPCR data, one-tailed Student's t-tests on biological replicates were used for single comparisons of treatment vs control. For ruthenium red staining, one replicate was performed with 10 to 20 plants per time point and treatment. For antibody staining assays, two replicates were performed for each time point and treatment. The quantifications from each replicate were pooled together and one-tailed Student's t-tests were used for single or multiple comparisons. The p-values for multiple comparisons were adjusted using the Bonferroni correction.

### Accession numbers

Sequence data from this article can be found in the GenBank/EMBL data libraries under accession numbers listed in Supplemental Table S2.

## Supplementary Material

kiad343_Supplementary_Data

## Supplemental data

The following materials are available in the online version of this article.

**Supplemental Figure S1.** *A. thaliana PMEs* and *PMEIs* are differentially expressed during haustorium development

**Supplemental Figure S2.** PME activity is increased during haustorium development

**Supplemental Figure S3.** *PjPMEs* and *PjPMEIs* are specific to haustoria

**Supplemental Figure S4.** *PjPME* and *PjPMEI* overexpression does not affect xylem connection to the host

**Supplemental Figure S5.** *A. thaliana* BR signalling mutants do not affect parasitism efficiency

**Supplemental Figure S6.** NAA treatment does not affect parasitism efficiency

**Supplemental Table S1.** List of identified PjPMEs and PjPMEIs

**Supplemental Table S2.** *P. japonicum* gene accession numbers and IDs

**Supplemental Table S3.** Primers used in the study

**Supplemental Video S1.** Confocal Z-stack of a haustorium expressing *PjPMEI9-2xVenus::NLS*
