# Supplementary material for: Pectin modifications promote haustoria development in the parasitic plant Phtheirospermum japonicum
Source: Plant Physiol. 2023 Jun 13;194(1):229–42. doi: 10.1093/plphys/kiad343 (PMC10762509; doi:10.1093/plphys/kiad343)
Supplement: kiad343_Supplementary_Data [file kiad343_supplementary_data.zip › SupplementalFigureS1-6.pdf]

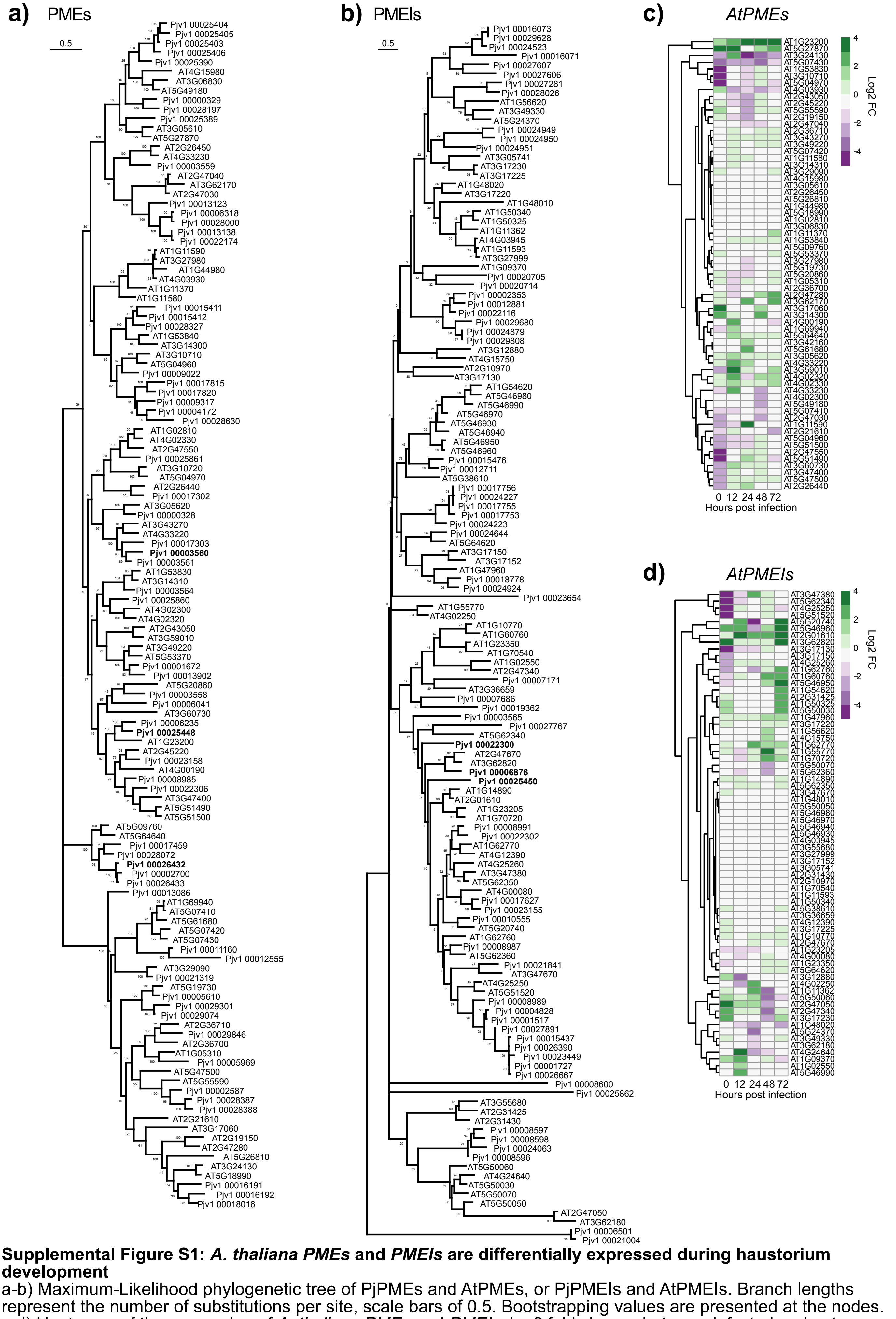

Supplemental Figure S1: *A. thaliana PMEs* and *PMEIs* are differentially expressed during haustorium development

a-b) Maximum-Likelihood phylogenetic tree of PjPMEs and AtPMEs, or PjPMEIs and AtPMEIs. Branch lengths represent the number of substitutions per site, scale bars of 0.5. Bootstrapping values are presented at the nodes. c-d) Heatmaps of the expression of *A. thaliana PMEs* and *PMEIs*: log2 fold change between infected and not infected *A. thaliana* over five time points during infection. The genes are clustered by expression profile.

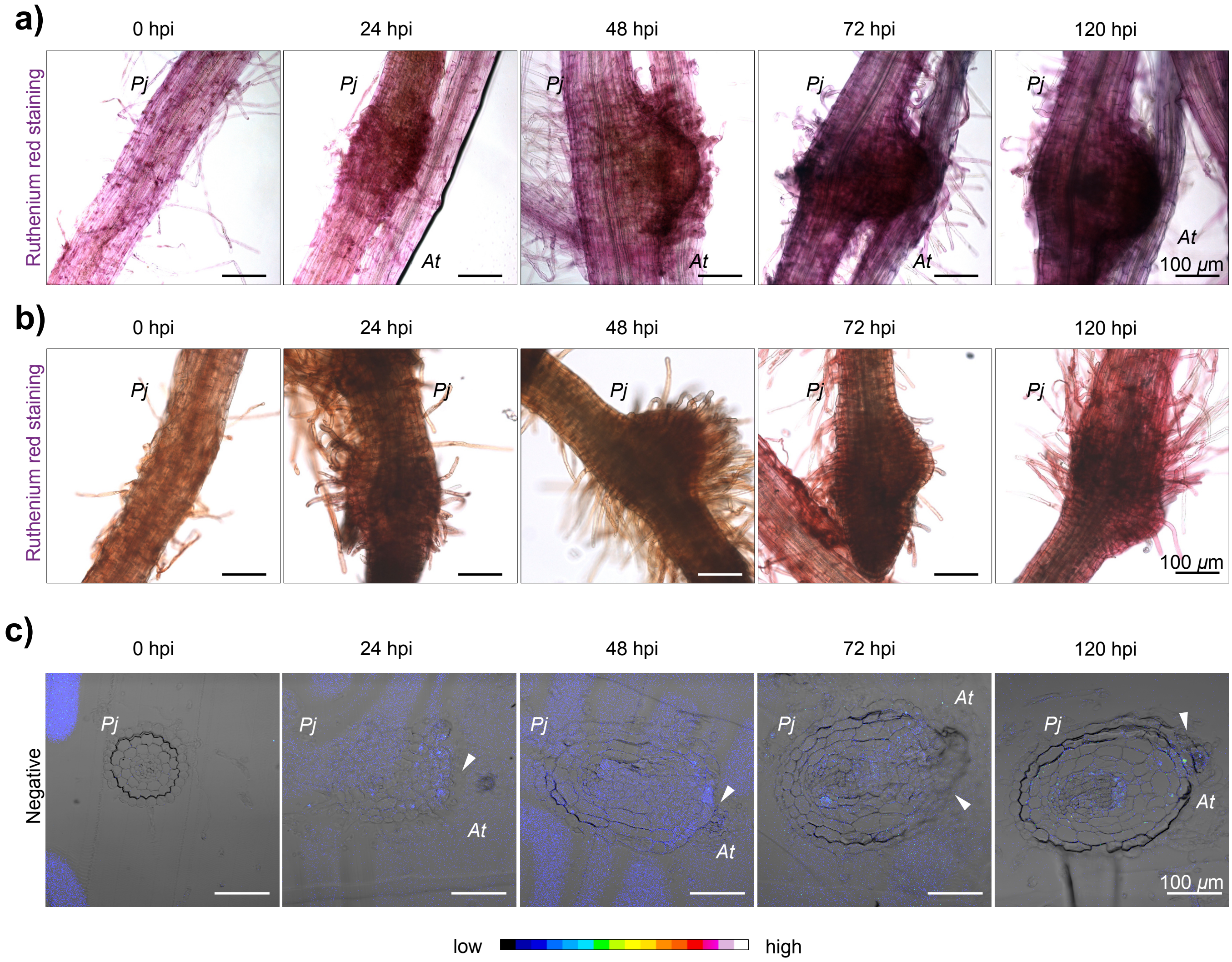

Supplemental Figure S2: PME activity is increased during haustorium development
a) Ruthenium red staining of the developing haustoria at 0, 24, 48, 72 and 120 hours post infection (hpi). 24, 48 and 120 hpi images are repeated from Fig. 2a b) Ruthenium red staining of the developing pre-haustorium at 0, 24, 48, 72 and 120 hours post exposure to DMBQ. c) Fluorescence images of the antibody staining negative control (PBS) on haustoria cross section at 0, 24, 48, 72 and 120 hpi. Scale bars 100 μm; *Pj* = *P. japonicum*, *At* = *A. thaliana*; arrowheads point at the host-parasite interface.

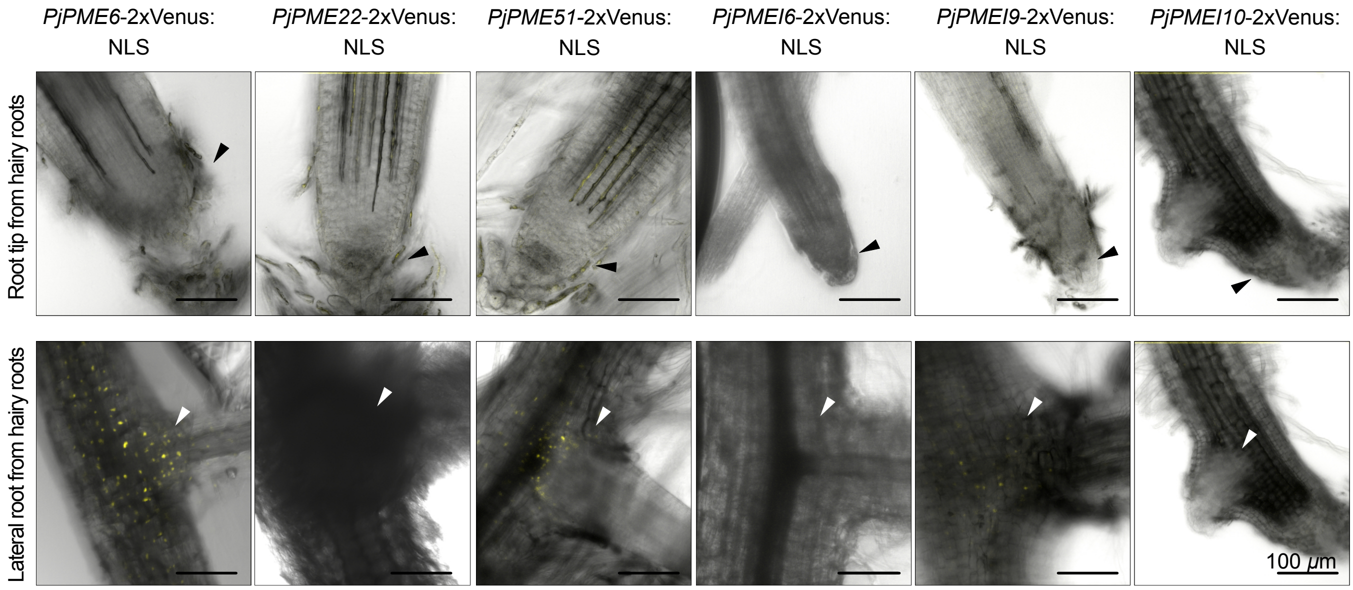

Supplemental Figure S3: *PjPMEs* and *PjPMEIs* are specific to haustoria

Images of transgenic hairy roots expressing *PjPME6*, *PjPME22, PjPME51, PjPMEI6*, *PjPMEI9* and *PjPMEI10* nuclear-localized transcriptional reporters: root tips and lateral roots. Scale bars 100 μm. Black arrowheads point at root tips, white arrowheads point at lateral root emergence sites. The same image is presented for the root tip and lateral root of the *PjPMEI10*-2xVenus:NLS hairy root.

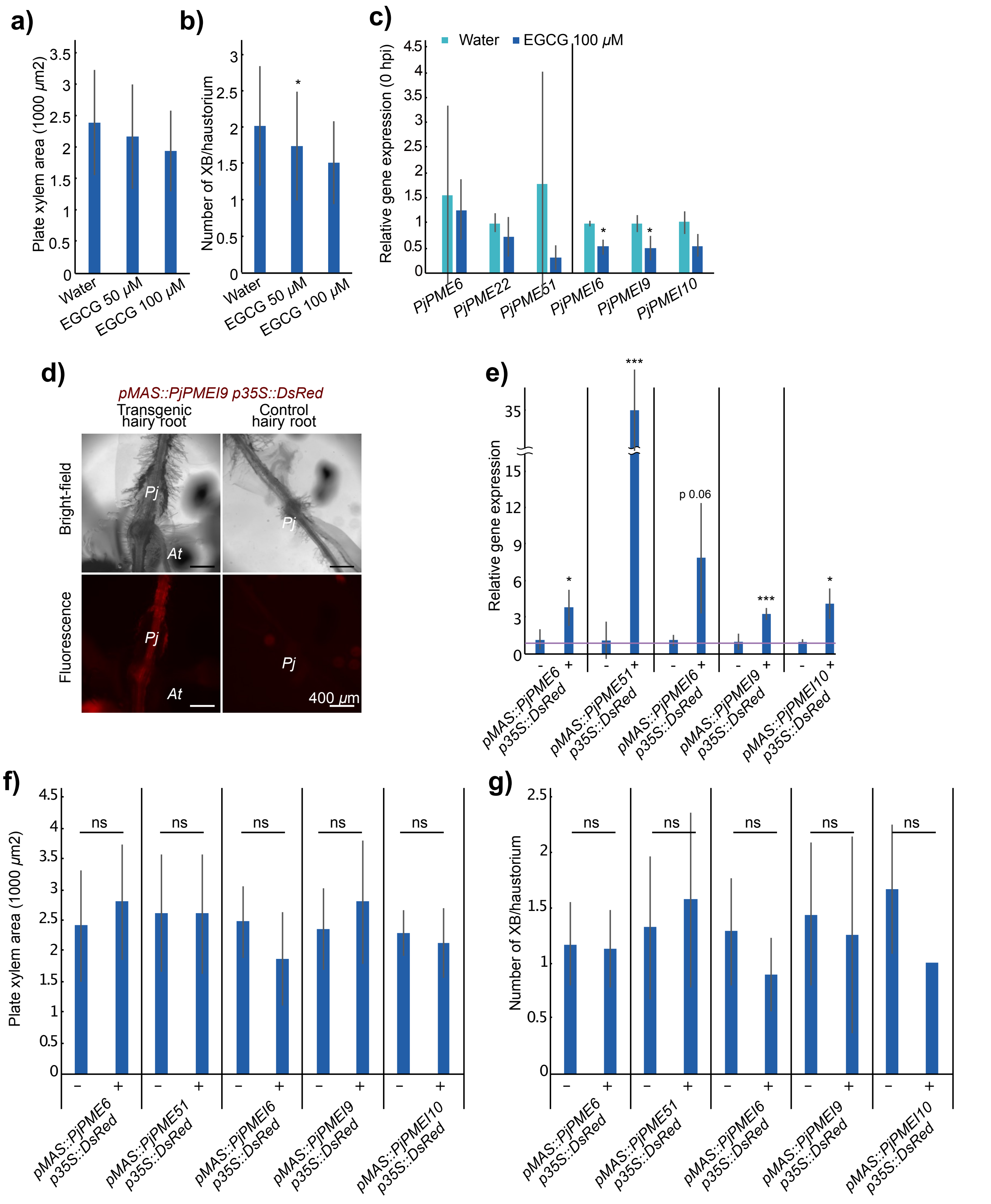

Supplemental Figure S4: *PjPME* and *PjPMEI* overexpression does not affect xylem connection to the host
a) Area of plate xylem in 7 dpi haustoria treated with 50 or 100 μM EGCG or water as control (n = 56-98 haustoria). b) Number of xylem bridges (XB) per haustorium in 7 dpi haustoria treated with 50 or 100 μM EGCG or water as control (n = 56-98 haustoria). c) Relative gene expression of selected *PjPMEs* and *PjPMEIs* at 0 hpi in *P. japonicum* haustoria treated with 100 μM EGCG, normalized to water (n = 3 biological replicates). d) Representative images of hairy roots transformed with gene overexpression constructs. Control hairy roots show no fluorescence, while transformed hairy roots have red fluorescence. *At* = *A. thaliana*, *Pj* = *P. japonicum*. Scale bar 400 μm. e) Relative gene expression of the *PjPME* or *PjPMEI* of interest in hairy roots transformed with the indicated construct (+) and control roots (-) (n = 3 replicates). f) Area of plate xylem in 7 dpi haustoria formed on hairy roots transformed with the indicated construct (+) and control roots (-) (n = 3-29 haustoria). g) Number of XB per haustorium in 7 dpi haustoria formed on hairy roots transformed with the indicated construct (+) and control roots (-) (n = 3-29 haustoria). For all panels, asterisks indicate significance compared to control (Student's t-test)* for $p<0.05$, ** for $p<0.01$, *** for $p<0.001$, bars represent standard deviation. ns = not significant at p=0.05.

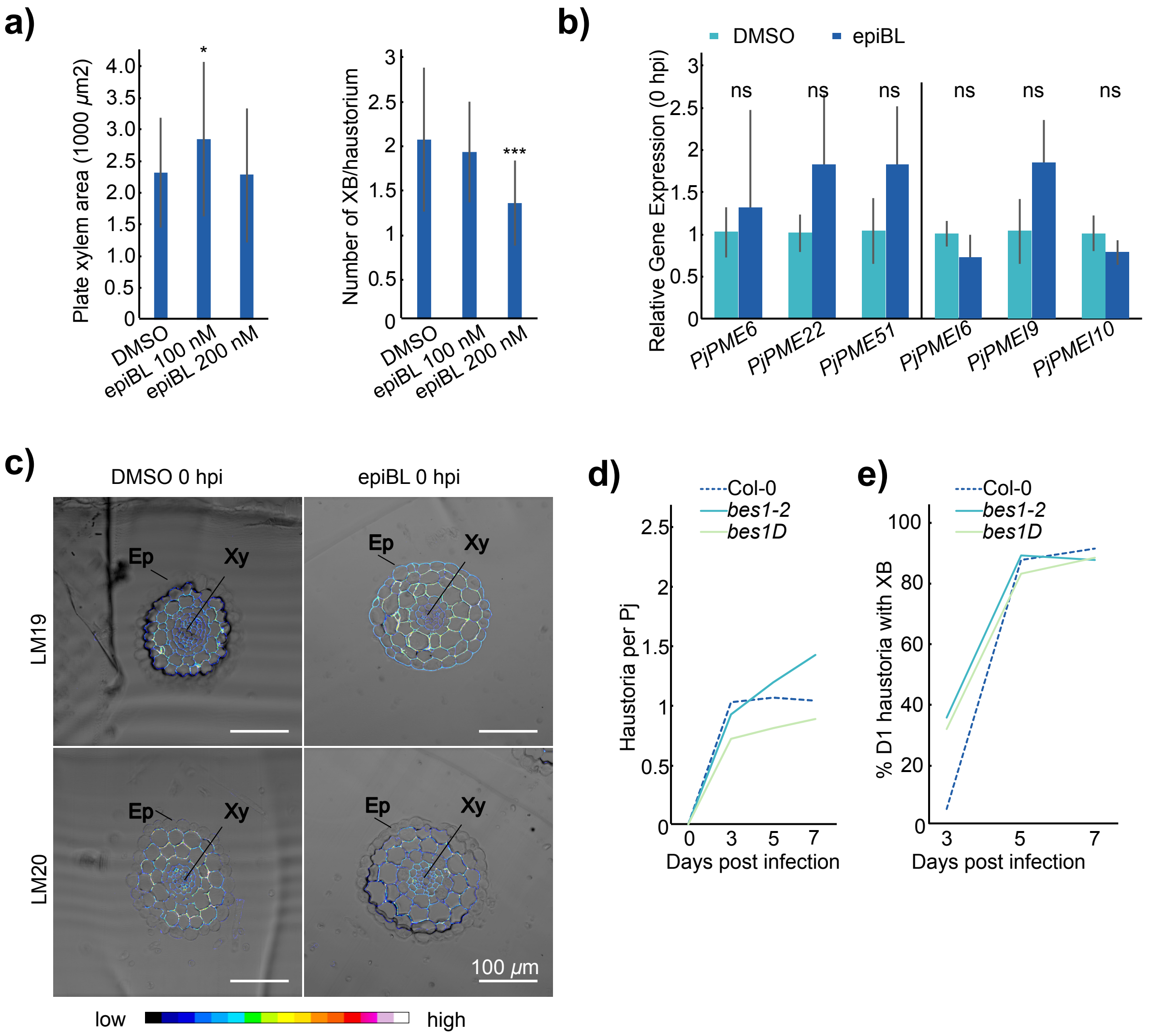

Supplemental Figure S5: *A. thaliana* BR signalling mutants do not affect parasitism efficiency
a) Area of plate xylem and number of xylem bridges (XB) per haustorium in 7 dpi haustoria treated with 100 nM and 200 nM epiBL or DMSO as control (n = 16-66 haustoria) b) Relative gene expression of selected *PjPMEs* and *PjPMEIs* at 0 hpi in *P. japonicum* haustoria treated with 100 nM epiBL, normalized to DMSO (n = 3 replicates). c) Fluorescence images of antibody staining using LM19 (unmethylated homogalacturonan) and LM20 (highly methylated homogalacturonan) on cross sections of haustoria developed on DMSO or 100 nM epiBL at 0 hpi. Scale bars 100 μm. Ep=epidermis, Xy=xylem. d) Number of haustoria per *P. japonicum* plant at four time points during infection of *bes1-2* and *bes1-D* mutants or Col-0 as control (n = 2 replicates). e) Percentage of Day-1 (D1) haustoria with a XB formed during infection of *bes1-2* and *bes1-D* mutants or Col-0 at three time points (n = 2 replicates). For all panels, asterisks indicate significance compared to control (Student's t-test): * for p<0.05, ** for p<0.01, *** for p<0.001, bars represent standard deviation. ns = not significant at p=0.05.

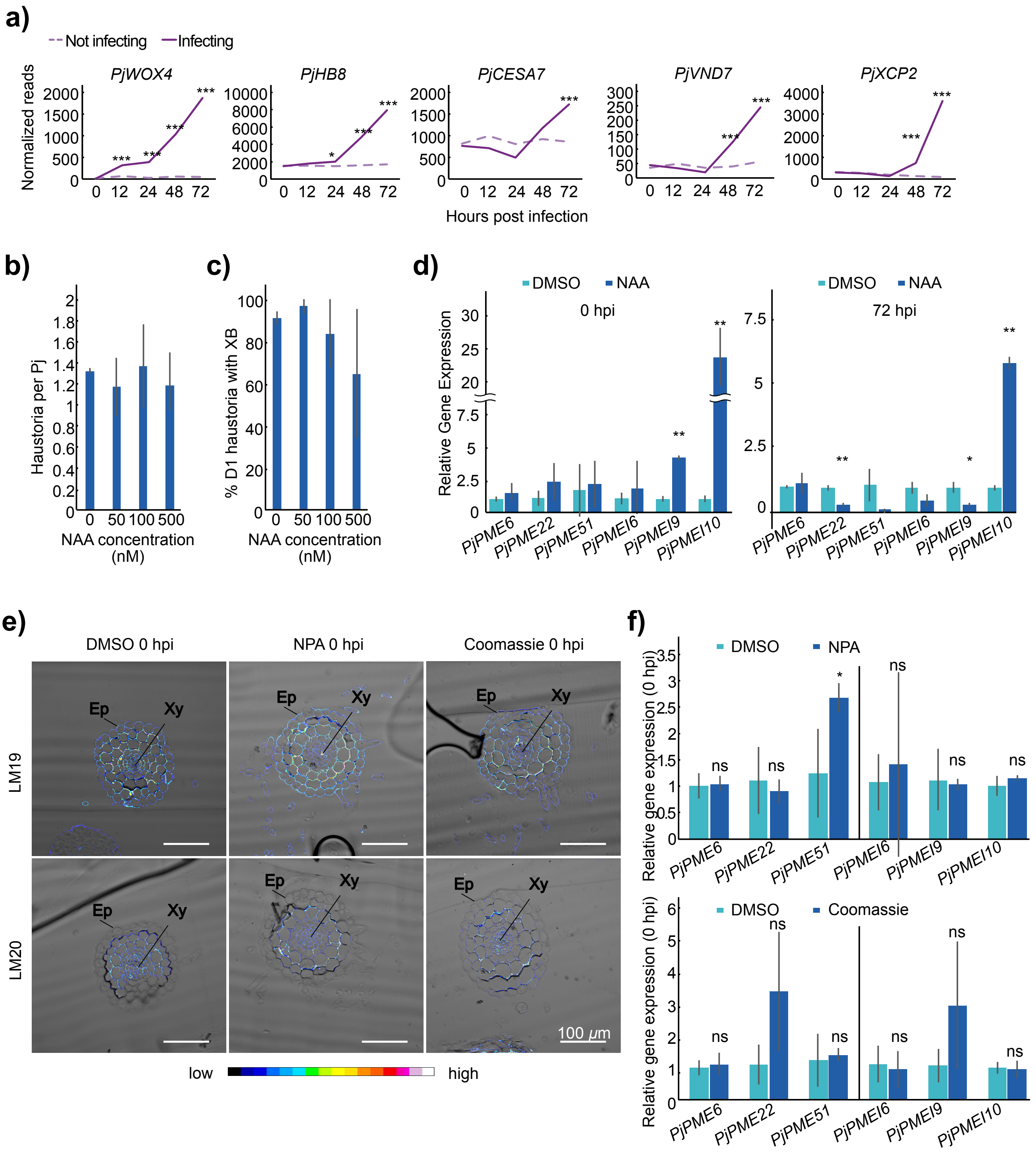

Supplemental Figure S6: NAA treatment does not affect parasitism efficiency
a) Normalized reads of *PjWOX4*, *PjHB8*, *PjCESA7*, *PjVND7* and *PjXCP2* over five time points during infection for *P. japonicum* infecting and not infecting. Asterisks indicate significant difference between infecting and not infecting (Wald test with Benjamini-Hochberg correction for multiple testing, n = 3 libraries) * for p<0.05, *** for p<0.001. b) Number of haustoria per *P. japonicum* plant at 7 dpi during treatment with 0, 50, 100 or 500 nM NAA (n = 3 replicates). c) Percentage of Day-1 (D1) haustoria with a xylem bridge (XB) formed during treatment with 0, 50, 100 or 500 nM NAA (n = 3 replicates). d) Relative gene expression of selected *PjPMEs* and *PjPMEIs* at 0 and 72 hours post infection in *P. japonicum* haustoria treated with 1 μM NAA, normalized to DMSO (n = 3 replicates). e) Fluorescence images of antibody staining using LM19 (unmethylated homogalacturonan) and LM20 (highly methylated homogalacturonan) on cross sections of 0 hpi haustoria developed on DMSO, 5 uM NPA or 0.05 mM Coomassie + DMSO. Scale bars 100 μm. Ep=epidermis, Xy=xylem. f) Relative gene expression of selected *PjPMEs* and *PjPMEIs* at 0 hpi in *P. japonicum* haustoria treated with 5 μM NPA or 0.05 mM Coomassie, normalized to DMSO (n = 3 biological replicates). For panels b to f, Asterisks indicate significance compared to control (Student's t-test) * for p<0.05, ** for p<0.01, bars represent standard deviation. ns = not significant at p=0.05.
