# Supplementary material for: Pectin modifications promote haustoria development in the parasitic plant Phtheirospermum japonicum
Source: Plant Physiol. 2023 Jun 13;194(1):229–42. doi: 10.1093/plphys/kiad343 (PMC10762509; doi:10.1093/plphys/kiad343)
Supplement: kiad343_Supplementary_Data [file kiad343_supplementary_data.zip › SupplementalMovieLengends.pdf]

Supplemental Video S1

Video of Z-stack images from a 4 days-post-infection haustorium formed by a *P. japonicum* transgenic hairy root expressing the *PjPMEI9* nuclear-localised (NLS) transcriptional reporter (yellow signal). 35 images were acquired at 2.88 um intervals. *Pj* = *Phtheirospermum japonicum*; *At* = *Arabidopsis thaliana*. Scale bar = 100 um.
